# Electrospun bioresorbable polymer membranes for coronary artery stents

**DOI:** 10.3389/fbioe.2024.1440181

**Published:** 2024-08-21

**Authors:** Maria A. Rezvova, Evgeny A. Ovcharenko, Kirill Yu Klyshnikov, Tatiana V. Glushkova, Alexander E. Kostyunin, Daria K. Shishkova, Vera G. Matveeva, Elena A. Velikanova, Amin R. Shabaev, Yulia A. Kudryavtseva

**Affiliations:** Research Institute for Complex Issues of Cardiovascular Diseases, Kemerovo, Russia

**Keywords:** coronary covered stents, percutaneous coronary intervention, perforation, stents, aneurysm, in-stent restenosis, bioresorbable polymers

## Abstract

Percutaneous coronary intervention, a common treatment for atherosclerotic coronary artery lesions, occasionally results in perforations associated with increased mortality rates. Stents coated with a bioresorbable polymer membrane may offer an effective solution for sealing coronary artery perforations. Additionally, such coatings could be effective in mitigating neointimal hyperplasia within the vascular lumen and correcting symptomatic aneurysms. This study examines polymer membranes fabricated by electrospinning of polycaprolactone, polydioxanone, polylactide-co-caprolactone, and polylactide-co-glycolide. In uniaxial tensile tests, all the materials appear to surpass theoretically derived elongation thresholds necessary for stent deployment, albeit polydioxanone membranes are found to disintegrate during the experimental balloon expansion. As revealed by *in vitro* hemocompatibility testing, polylactide-co-caprolactone membranes exhibit higher thrombogenicity compared to other evaluated polymers, while polylactide-co-glycolide samples fail within the first day post-implantation into the abdominal aorta in rats. The PCL membrane exhibited significant water leakage in the permeability test. Comprehensive evaluation of mechanical testing, bio- and hemocompatibility, as well as biodegradation dynamics shows the advantage of membranes based on and the mixture of polylactide-co-caprolactone and polydioxanone over other polymer groups. These findings lay a foundational framework for conducting preclinical studies on stent configurations in large laboratory animals, emphasizing that further investigations under conditions closely mimicking clinical use are imperative for making definitive conclusions.

## 1 Introduction

Coronary Artery Disease (CAD) is currently recognized as one of the leading causes of mortality globally, representing a significant prevalence among cardiovascular disorders. The genesis of CAD is often linked to atherosclerotic diseases affecting the coronary arteries (Iqbal et al., 2013). Percutaneous Coronary Intervention (PCI), an established therapeutic approach for CAD, is performed more than two million times annually, and the use of the procedure keeps rising around the world (Abubakar et al., 2023). In a subset of cases (approximately 0.7%), interventions such as balloon angioplasty or stent implantation can provoke Coronary Artery Perforations (CAP) that is a relatively infrequent but serious complication associated with elevated mortality risks (Avula et al., 2022; Kuno et al., 2023). This highlights the critical necessity for effective and accurate treatment approaches.

Stents covered with a polymer membrane have emerged as an effective strategy for addressing CAP (Lemmert et al., 2017). This method of reconstruction does not require open surgical operations significantly reducing trauma (Al-Mukhaini et al., 2011). The polymer membranes form a barrier impermeable to blood facilitating the effective closure of perforations and preventing neointimal growth within the vascular lumen (Lemmert et al., 2017). Expanded polytetrafluoroethylene (ePTFE) is one of the potential materials for coating coronary stents in CAP therapy (Briguori et al., 2000), and there has been notable progress achieved in sealing CAP with such devices (Kufner et al., 2019). However, the use of stent-grafts like Graftmaster (Jostent) featuring an ePTFE layer encased between two metallic stent frames is constrained due to their considerable size and limited flexibility. This limitation renders them less effective for use in the narrower, more remote segments of small-diameter arteries (Gruberg et al., 2000). Moreover, there are concerns about the risk of thrombosis associated with these devices which requires the implementation of dual antiplatelet therapy (Kwok et al., 2001; Wang et al., 2017). It is known that stents covered with a biological material membrane, such as equine pericardium, can improve flexibility and minimize thrombogenicity (Chen et al., 2015), but long-term implantation have indicated instances of restenosis (Secco et al., 2016). When stents are placed in vessels for prolonged periods, xenogenic tissues may also trigger inflammatory responses and calcification (Agathos et al., 2019).

A significant advancement beyond previously described stent membranes is represented by the FDA-approved PK Papyrus stent (Biotronik AG) which is coated with a material based on polyurethane. It is its thickness that distinguishes this advanced device in the context of sealing coronary artery perforations (CAP). The stent features a 90 µm thin membrane fabricated through electrospinning onto a metallic frame of merely 60 µm in thickness which endows the implant with both flexibility and compactness (Kandzari and Birkemeyer, 2019). However, the selection of biostable polyurethane as the stent membrane material over a long term may lead to restenosis due to inflammatory reactions to the foreign body or infections. In this context, the utilization of a biodegradable polymeric material is preferable. Bioresorbable synthetic membranes prepared through electrospinning from polylactide, polycaprolactone, and their copolymers, have demonstrated favorable outcomes in animal studies targeting the treatment of vascular aneurysms (Wu et al., 2014), preventing neointimal growth due to drug loading (Zhu et al., 2013; Kuznetsov et al., 2020; Chausse et al., 2023), and decreasing thrombosis due to the hydrophilicity of the polymeric membrane (Boodagh et al., 2016). However, for translating this experience into the treatment of CAP, properties of the membranes such as permeability, stretchability, stiffness, structural changes upon balloon expansion, hemocompatibility, compatibility with blood cells and components, as well as biodegradation, need to be investigated.

To address the discussed limitations, bioresorbable electrospun membranes based on polycaprolactone (PCL), polydioxanone (PDO), polylactide-co-caprolactone (PLCL), and polylactide-co-glycolide (PLGA) were studied *in vitro* and *in vivo*. The functionality of a prototype of the covered coronary stent was evaluated during crimping and balloon expansion. For reference values, we adopted characteristics demonstrated by a successful *in vivo* study of the PK Papyrus stent: membrane thickness is approximately 90 μm when fully expanded, profile thickness is 60 μm, fiber size is approximately 2 μm, and maximum expansion ranges from 2.5 to 4 mm (Kandzari and Birkemeyer, 2019). The key advantages of the investigated stent-membranes include their thin profile, the ability to significantly change diameter during balloon expansion, enabling the device to remain compact and mobile, as well as degradation over time.

## 2 Materials and methods

Poly (ε-caprolactone) (PCL) with an average molecular weight (M_n_) of 80,000 (Sigma Aldrich, United States); polydioxanone (PDO) (Sigma Aldrich); poly (lactic-co-caprolactone) (PLCL) with a lactide to caprolactone ratio of 70:30 (Corbion, the Netherlands); poly (lactic-co-glycolic acid) (PLGA) with a molecular weight (M_w_) ranging from 30,000 to 60,000 and a lactide to glycolide ratio of 50:50 (Sigma Aldrich). Chloroform (CHCl_3_), ≥99.8% (Vecton, Russia), hexafluoro-2-propanol (1,1,1,3,3,3-hexafluoro-2-propanol, HFP), ≥99.8% (Sigma Aldrich). As a control material, Gore-Tex polymer membrane (Cardiovascular Patch GORE-TEX^®^, United States) fabricated from expanded polytetrafluoroethylene (ePTFE) was investigated. In the study, we used samples of a coronary bare metal stent of our own design with a thickness of about 70 µm. The samples were fabricated using laser cutting (MFT 120 Laser cutting machine, Swiss Tec AG, Switzerland) from a CoCr L605 alloy tube.

### 2.1 Electrospinning. Membrane characterization. Scanning electron microscopy

The membranes were fabricated via electrospinning using a Nanon-01A (MECC Inc., Japan) with an applied voltage of 25 kV and a solution feeding rate of 0.5 mL/h. A metallic pin rotating at 100 rpm with a diameter of 8 mm was employed as the receiving collector. The distance from the polymer jet outlet to the collector was set at 15 cm. All experiments were conducted at room temperature with an approximate relative humidity of 30%. Solution concentrations, solvent types, and membrane fabrication times were empirically optimized based on the desired thickness (80–100 µm) and fiber formation quality. The polymer membranes obtained in this manner were tested in all experiments except for balloon expansion, for which covered stents were prepared (Section 2.5).

Surface morphology assessment was performed using scanning electron microscopy (SEM). The samples were mounted on SEM stubs and sputter-coated with a gold-palladium layer using an Emitech SC 7640 vacuum post (Quorum Technologies, England) to enhance conductivity. Observations were made with an S-3400N scanning electron microscope (Hitachi, Japan) under high vacuum at an accelerating voltage of 10 kV in secondary electron mode.

### 2.2 Contact angle assessment

The water contact angle was determined using the “sessile drop” method on experimental equipment. A 15 µL droplet of distilled water was placed onto the flat surface of the samples and stabilized for 5 s for each measurement. All tests were conducted at room temperature. The obtained images were processed using the Contact Angle plugin in the ImageJ software program (National Institutes of Health, Bethesda, MD, United States). The analysis was repeated 8 times for each sample group.

### 2.3 Mechanical characterization

The mechanical properties of the obtained materials were evaluated under uniaxial tension according to ISO 37:2017 at the temperature of 37°C. Test samples were prepared using a ZCP 020 cutting press (Zwick GmbH and Co. KG, Germany) and a specially shaped knife (B083, as per ISO 37:2017 standard), n = 7–8. The direction was not considered due to the isotropic nature of the material properties. The conclusion about the isotropy of the materials was drawn based on the results of preliminary experiments. Investigations were conducted on a Z series universal testing machine (Zwick GmbH and Co. KG) equipped with a sensor having a nominal force of 50N. The crosshead speed was set at 50 mm/min. The tensile strength of the material was determined by evaluating the maximum stress during stretching (in MPa), factoring in the cross-sectional area of the sample. The elastic-deformative properties were assessed based on relative elongation adjusted for the nature of sample failure (in %) and the Young’s modulus which was calculated over the elongation range necessary to produce the final product (from 0% to 125% elongation). Sample thickness measurements were performed using a thickness gauge, which has a permissible error limit of ±0.01 mm and a clamping force not exceeding 1.5N.

### 2.4 Testing the deployment of stents covered with polymeric membrane

To form a polymer coating on the stent, the stent was mounted on a pin with a diameter of 1.5 mm. To prevent stent rotation on the pin during the electrospinning process, it was crimped using an experimental equipment for radial compression. The outer diameter of the metal stent was 1.8 mm.

Coatings of the investigated polymers (PCL, PLCL, PDO, PLCL/PDO, PLGA) were formed on the outer surface of the stent using the electrospinning method. After obtaining samples of stents coated with various membranes, these samples were crimped onto a commercially available medical balloon with a working length of 30 mm for inflating coronary stents “Driver” (Medtronic Plc., United States) (Figure 3) using an experimental equipment.

Subsequently, stent expansion with the balloon was performed using a standard high-pressure syringe inflator until the nominal outer diameter of the stent-graft reached 4.0 mm. The pressure was then reduced, and the balloon was removed. The structure of the polymer coating before and after balloon expansion was evaluated using SEM.

### 2.5 Cytocompatibility assessment

The Ea. hy926 cell line was chosen as the experimental endothelial cell line. This represents a hybridoma line derived from human endothelium and A549/8 cells. Cells were cultivated in DMEM/F12 nutrient medium (11,320,033, Thermo Fisher Scientific, United States) supplemented with HAT (H0262, Sigma Aldrich), 10% fetal bovine serum (26,140,079, Thermo Fisher Scientific), antibiotics (0,378,016, Thermo Fisher Scientific), and amphotericin B (15,290,018, Thermo Fisher Scientific). The culture was passaged upon reaching 70% confluency, and cells were detached using a 0.025% trypsin-EDTA solution (15,400,054, Thermo Fisher Scientific). Experiments were conducted under sterile conditions, and cells were cultivated in a CO_2_-incubator with 5% CO_2_ content and elevated humidity. Ea. hy926 culture cells were seeded on fixed matrix samples at 50,000 cells/well and cultivated in complete nutrient medium for 3 days with media changes on days 1 and 3. The control group consisted of 24-well plate samples without matrices, where a corresponding number of cells were seeded and cultivated under similar conditions. After 3 days, cell viability was evaluated using fluorescent microscopy (n = 4, for each material type), metabolic activity through colorimetric methods (n = 6), and proliferative activity using fluorescent confocal microscopy (n = 4).

#### 2.5.1 Cell viability

Cells were stained with Hoechst 33,342 nuclear dye (10 μg/mL, 14,533, Sigma Aldrich) for 10 min and with ethidium bromide (30 μg/mL, 46,067, Sigma Aldrich) for 1 min. Cell counts on samples and culture plastic were carried out on an inverted microscope Axio Observer Z1 (Carl Zeiss, Germany) with 5 random fields of view from each replicate. For Hoechst 33,342, a wavelength of 350/461 was used, and a suitable one was used for ethidium bromide. A recalculation of cells from the field of view to S = 1 mm^2^ was conducted.

The relative number of dead cells was calculated using the formula: absolute number of dead cells*100%/absolute number of all adhered cells. The relative number of living cells was determined by subtracting the proportion of dead cells from 100% of adhered cells.

#### 2.5.2 Cell proliferation

Cell proliferative activity was assessed using the Click-iT™ Plus EdU Cell Proliferation Kit for Imaging (C10637, Thermo Fisher Scientific). Cells were incubated with the EdU reagent for 16 h, then stained according to the manufacturer’s instructions. After the procedure was completed, cells were further stained with the DAPI nuclear dye (10 μg/mL, D9542, Sigma Aldrich) for 30 min. Preparations were analyzed using the LSM700 scanning confocal microscope (Carl Zeiss). Ten randomly selected fields of view were evaluated from each sample at a 200 × magnification, with 2 samples for each polymer type. Quantitative image analysis was conducted using the ImageJ software, counting the total number of cells and the number of proliferating cells in the field of view. The relative number of proliferating cells was assessed using the formula: number of proliferating cells in the field of view *100/total number of cells in the field of view.

#### 2.5.3 Cell metabolic activity

Cell metabolic activity was assessed using a colorimetric method with the Cell Cytotoxicity Assay Kit—Colorimetric (ab112118, Abcam, United States). The reagent, at a working concentration (1:5 with the nutrient medium), was added to the wells containing the samples and incubated for 3 h at 37°C. Afterward, 200 µL of the reagent from the wells with samples was transferred to the wells of a 96-well plate and the optical density was measured at two wavelengths, 570 nm and 605 nm, using the Multiskan Sky spectrophotometer (Thermo Fisher Scientific).

### 2.6 Platelet adhesion

To evaluate platelet adhesion and the transformation level of adhered platelets, polymer material samples were incubated in platelet-rich plasma. This plasma was sourced from fresh citrated donor blood and obtained by centrifuging for 10 min at 1,200 rpm. Post-incubation, the samples were washed, fixed in a 2% glutaraldehyde solution, and then dehydrated through an ascending series of alcohol concentrations. Subsequently, a conductive coating of Au/Pd was applied to the sample surfaces using the EM ACE200 vacuum apparatus (Leica Mikrosysteme GmbH, Austria). The surface morphology of the polymer materials after contact with platelets, was examined using scanning electron microscope under high vacuum conditions and an accelerating voltage of 15 kV. The adhesive capability of the polymer surfaces was assessed based on the deformation index (DI) of the platelets, the number of platelets per 1 mm^2^, and the predominant type of platelet. The deformation index was computed using the formula: DI = (Number of Type I × 1 + Number of Type II × 2 + Number of Type III × 3 + Number of Type IV × 4 + Number of Type V × 5)/total number of platelets.

### 2.7 Hemocompatibility *in vivo*

*In vivo* experiments were conducted on male Wistar rats weighing 350–400 g. All surgical procedures were performed under inhalation anesthesia with isoflurane in a sterile operating room environment. Polymer membrane samples (PCL, PLCL/PDO, PLGA, and ePTFE) were pre-sterilized with ethylene oxide and placed in a sterile 0.9% NaCl solution before implantation.

**FIGURE 1:**
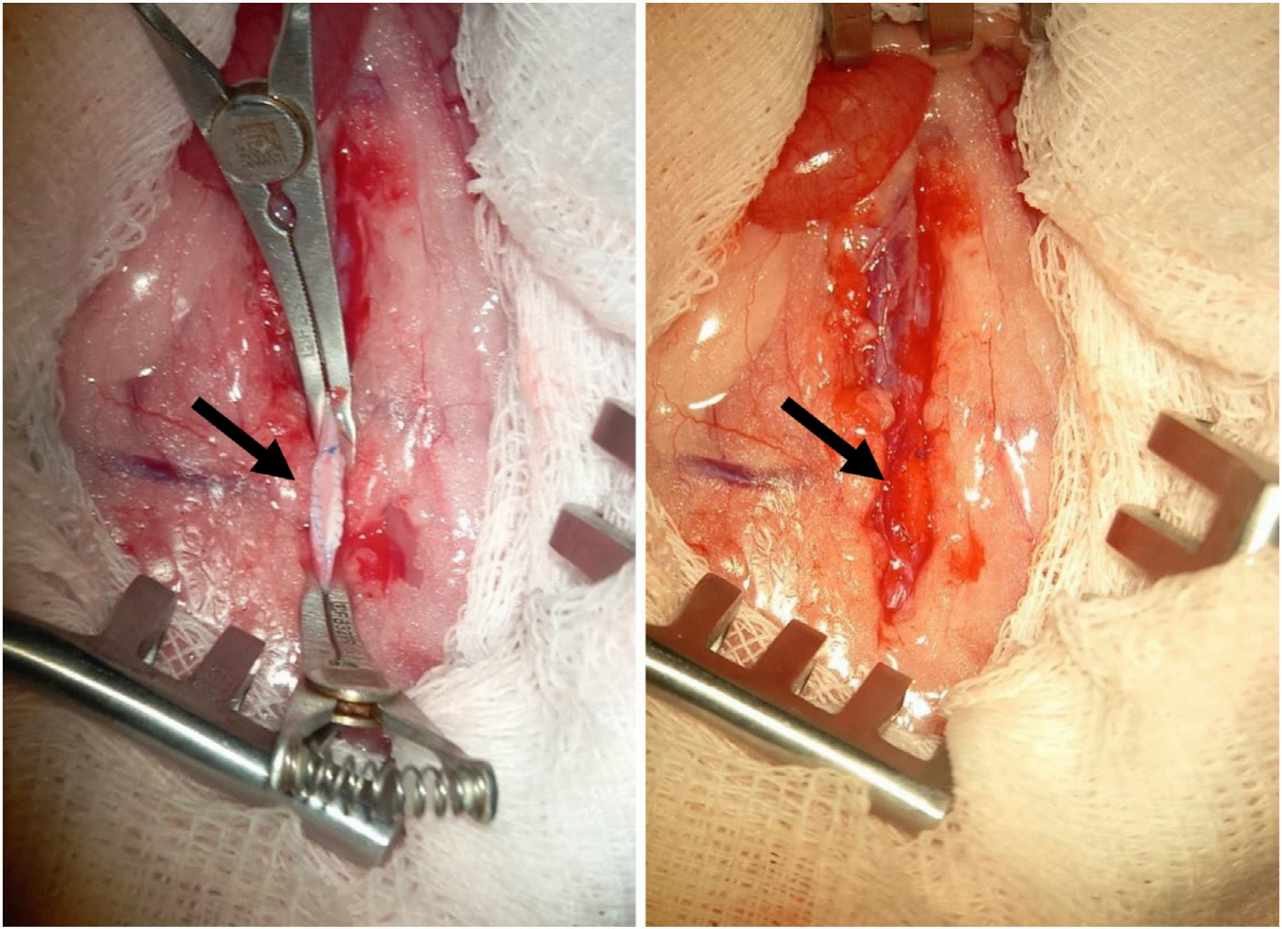
An example of a membrane made of polymer material (indicated by the arrow), implanted into the position of the rat abdominal aorta.

Before performing the surgery, the fur on the abdomen of the animals was carefully shaved, and the implantation area was treated with a skin antiseptic. After a midline incision was made on each animal’s abdomen to gain access to the abdominal aorta. Following this, a longitudinal incision was made in the aortic wall, and a polymer patch measuring 2 × 5 mm^2^ was sewn in to form the anterior wall of the vessel (Figure 1). At the final stage of the operation, the abdominal wall was sutured layer by layer using non-absorbable polyester suture material, Lavsan 4.0 (Lintex). Each polymer material was implanted for a duration of 5 and 20 days (5 animals per time point). Upon completion of the experiment, the animals were euthanized using carbon dioxide gas, and the polymer patches were removed for microscopic analysis along with the aorta and adjacent perivascular adipose tissue.

During the experimental studies involving laboratory animals, the principles of the European Convention (Strasbourg, 1986) and the Helsinki Declaration of the World Medical Association regarding the humane treatment of animals (1996) were followed. The conduct of experiments on small laboratory animals was approved by the Local Ethics Committee of the Research Institute for Complex Issues of Cardiovascular Diseases, Kemerovo (protocol No. 08/1, approval date: 08/27/2021).

#### 2.7.1 Histological and immunohistochemical staining

Samples of polymer membranes and surrounding tissues extracted from the rat aortas were rinsed in a 0.9% NaCl solution and fixed in Neg-50 tissue freezing medium (6,502, Thermo Fisher Scientific) for rapid tissue freezing, followed by placement in liquid nitrogen. Cross-sections of the aorta (at the site of membrane implantation) with a thickness of 6 µm were prepared using a cryostat microtome HM525 (Thermo Fisher Scientific) and mounted on glass slides.

Structural changes in the polymer materials and their integration with surrounding tissues were assessed using Movat-Russell Pentachrome staining. For this purpose, sections were fixed in 4% paraformaldehyde for 10 min and then washed three times (5 min each) in distilled water on a shaker. Subsequently, the sections were subjected to staining using reagents from a commercial kit for Movat-Russell Pentachrome staining according to the manufacturer’s protocol (ab245884, Abcam). Upon completion of staining, the sections were covered with cover glass using mounting medium Vitrogel (BioVitrum, Russia).

The intensity of the inflammatory response following the implantation of the studied polymeric membranes was assessed using immunohistochemical staining with antibodies against the pan-leukocyte marker CD45 (ab10558, Abcam), the macrophage marker CD68 (ab125212, Abcam), and neutrophil myeloperoxidase MPO (ab208670, Abcam). Prior to staining, the sections were fixed for 10 min at room temperature in 4% paraformaldehyde, followed by three washes (5 min each) in phosphate-buffered saline with a pH of 7.4 on a shaker. The immunohistochemical reaction was carried out using the NovoLink Polymer Detection System (Leica Microsystems Inc.) according to a modified protocol provided by the manufacturer. Initially, endogenous peroxidase activity was blocked with a 4% hydrogen peroxide solution (Peroxidase Block) for 5 min. Subsequently, the sections were washed twice in phosphate-buffered saline and nonspecific antibody binding was blocked with a 0.4% casein salt solution containing auxiliary reagents (Protein Block) for an hour. Primary antibodies were diluted according to the manufacturer’s protocol in a 1% bovine serum albumin saline solution at a ratio of 1:1,000. The sections were incubated with the antibodies for 20 h in a closed box at + 4°C, followed by three washes in phosphate-buffered saline and a 30-min incubation with secondary anti-rabbit antibodies (Novolink Polymer). After three washes in phosphate-buffered saline, the sections were treated with a 0.087% diaminobenzidine solution for 2 min, washed in distilled water for 5 min, and placed in hematoxylin (from the kit) for 10 min. This was followed by bluing the sections in running water for 5 min, dehydrating in three changes of 95% ethanol (5 min each), clearing in three changes of xylene (5 min each), and finally mounting under a cover slip using Vitrogel mounting medium (BioVitrum).

The preparation of images of the stained samples was performed using an automated laboratory biological microscope MT5300L (Meiji Techno, Japan). Image processing was carried out with the Vision Slide software. Measurements of neointima thickness and thrombotic masses, as well as the density of positively stained cells, were conducted using the QuPath software version 0.4.1. The area of interest for cell counting was limited to the section of the polymeric sample and the surrounding peri-implantation space, equal to 200 μm. For these measurements, two sections from each sample were selected.

### 2.8 Permeability assessment

The water permeability test was performed in compliance with the ISO 7198:2016 standard. Six different polymers were tested: PCL, PDO, PLCL, PLCL/PDO, PLGA, and ePTFE (control). For each polymer type, ten samples were prepared: five in their initial, unstretched state, and five subjected to uniaxial deformation of 125%, simulating the radial deformation experienced by implanted stent grafts. Water at room temperature was used with a pressure of 120 ± 2 mmHg.

The total leakage and permeability were collected and reported as mean ± SD. Permeability was calculated using the (Formula 1):

$$W=Q/\left(A\;*\;t\right)$$ (1)

where W is the permeability (mL/min/mm^2^), Q–is the volume of the fluid passing through the membrane (mL), A–is the working area of the membrane (50 mm^2^), t–is the testing time (10 min).

Data is presented in terms of 1 mm^2^ as this is the approximate area of potential perforation.

### 2.9 Statistical analysis

Statistical analysis was conducted using GraphPad Prism 8.0 (GraphPad Software, version 8.00, San Diego, CA, United States). The normality of distribution was checked using the Shapiro-Wilk test. The statistical significance of differences between groups was determined based on variance analysis using Fisher’s parametric test and *post hoc* comparison. Differences between the groups were assessed using the nonparametric Kruskal–Wallis test with Dunn’s correction for multiple comparisons. When distribution was normal, results were presented as the mean and standard deviation of the mean; for non-normal distribution, they were represented as the median, 25th, and 75th percentiles [Me (25%; 75%)]. A *p*-value of < 0.05 was considered statistically significant.

## 3 Results and discussion

### 3.1 Membrane characterization. Scanning electron microscopy

A significant number of contemporary publications is dedicated to the development of small-diameter vascular prostheses fabricated through electrospinning of bioresorbable synthetic polymers (Han et al., 2011; Rickel et al., 2021; Zavan et al., 2021; King and Bowlin, 2022; Weekes et al., 2022). The experience gained by researchers in this field has enabled the assessment of the potential suitability of various materials for forming a polymer membrane on coronary stents. The selection criteria for polymers in this study are high biocompatibility, satisfactory degradation rate, mechanical properties, commercial availability, and suitability for electrospinning. The following bioresorbable polymers were identified for functional characterization: PCL, PDO, PLCL, PLGA, and a composite material with fibers of PLCL and PDO.

**FIGURE 2:**
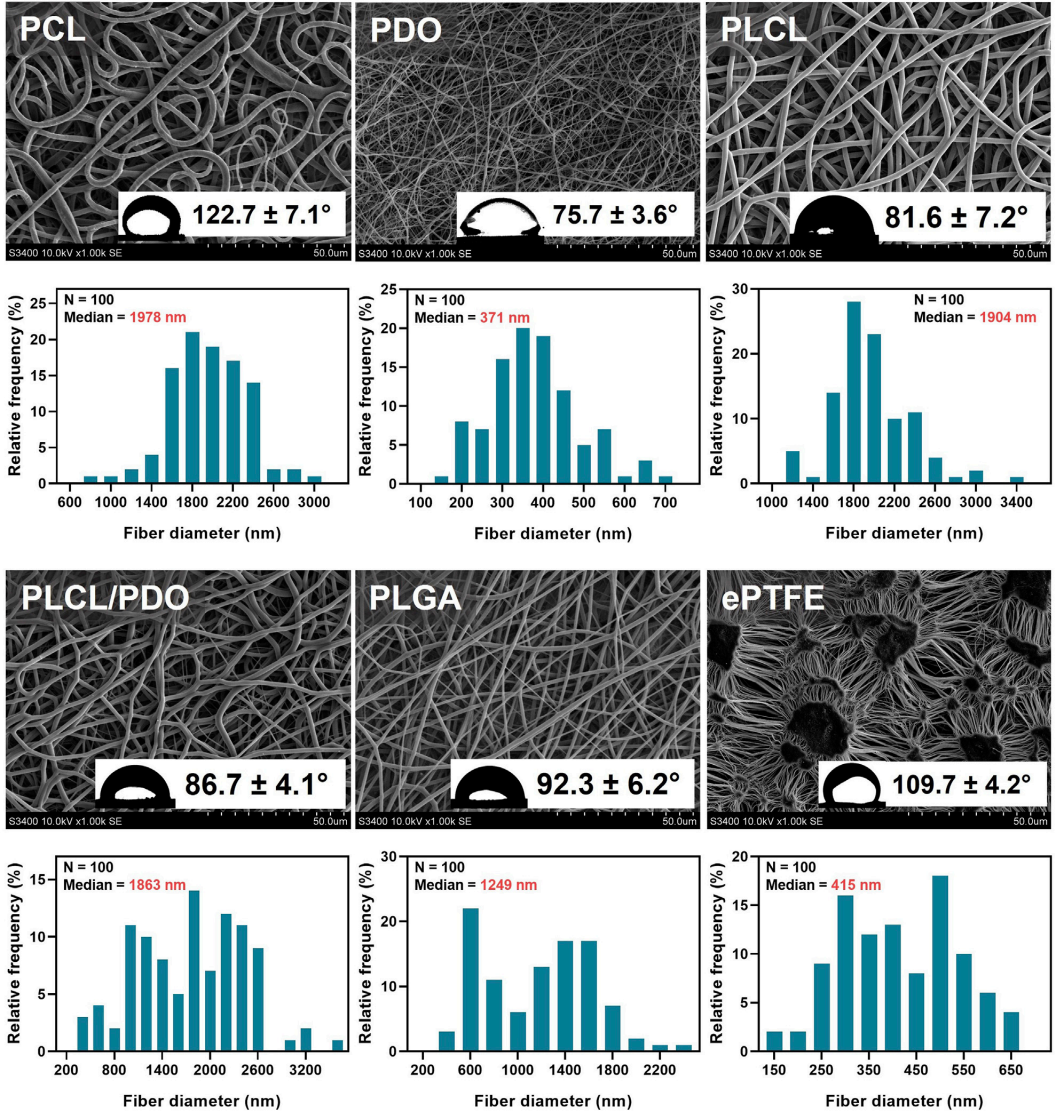
Structure analysis of electrospun polymer membranes PCL, PDO, PLCL, PLCL/PDO, and ePTFE investigated for coating coronary stents. For each type of polymers, the data shows scanning electron microscopy images of the membrane surface (magnification ×1,000), distribution of fibers by their diameter, and water contact angles obtained by the sessile drop method.

The electrospinning technique allows for creating structures of virtually any form contingent upon the collector type employed and thereby facilitates the deposition of thin tubular layers, including direct application onto the surface of a stent (Hu et al., 2012). Nevertheless, during the preliminary phase of this research, electrospinning was executed using a cylindrical collector of 8 mm in diameter to fine-tune the process modes and parameters. The selected operational modes (Table 1) were instrumental in generating a three-dimensional porous architecture characterized by uniform fibers across all investigated polymers (Figure 2). The concentration of stock solutions for each polymer type was determined based on preliminary experimental outcomes which demonstrated the absence of electrospinning defects, notably the formation of droplets (Rashid et al., 2021). Hexafluoroisopropanol (HFP) emerged as the optimal solvent for all materials except for polycaprolactone (PCL). As PCL membranes exhibited comparable fiber size and porosity when fabricated using either chloroform or hexafluoroisopropanol, the former was chosen due to its enhanced accessibility and reduced toxicity. The thickest fibers of 1,978 nm, 1,904 nm, and 1,863 nm were observed in membranes derived from PCL, PLCL, and PLCL/PDO, respectively (Figure 2). The finest fibers were produced using PDO measuring 371 nm in diameter. For comparison, Figure 2 also illustrates the porous configuration of the ePTFE polymeric material which is employed in clinically approved covered stents designed for the occlusion of coronary artery perforations.

**TABLE 1:** Parameters used in the fabrication of polymer membranes by electrospinning method.

Polymer	PCL	PDO	PLCL	PLCL/PDO	PLGA
Parameter					
Solvent	CHCl_3_	HFP			
Concentration, %	15	8	15	10/5	30
Manufacturing time	50 min	1 h 20 min	1 h 20 min	1 h 20 min	1 h
Membrane thickness, µm	∼100				

### 3.2 Contact angle assessment

The measurement of the static contact angle stands as one of the foremost techniques in examining wetting behavior of biomaterials and evaluating their potential for hemocompatibility. Reports suggest that hydrophilic materials facilitate reduced adhesion of protein molecules marking the initial phase in the intricate process of interaction between the implant and the blood system (Vogler, 2012). Moreover, hydrophilicity of a membrane is known to positively affect the adhesion and proliferation of cells (Pan et al., 2017). Nevertheless, a consensus on the optimal contact angle for the most hemocompatible biomaterial remains elusive as this parameter is influenced not only by the surface morphology but also by the chemical composition of the material, which can variably affect the adhesion of proteins and blood cellular components. Additionally, the specific requirements for material properties can differ based on the intended application. In the context of our study, preventing thrombotic complications and pathological tissue proliferation is imperative while cellular adhesion and proliferation are not necessary.

In the analysis of the membranes studied, apart from the comparative pairs PLCL/PDO and PLGA, and PLCL/PDO and PLCL, significant differences were observed (p < 0.05) in their contact angles. The ePTFE polymeric membrane, utilized as a control, falls into the hydrophobic category as evidenced by a water contact angle of 109.7° ± 4.2° which exceeds 90° (Figure 2). Similarly, the PCL (122.7° ± 7.1°) and PLGA (92.2° ± 6.2°) membranes were identified as hydrophobic. Conversely, the remaining polymeric membranes under investigation are classified as hydrophilic with contact angles below 90° (Figure 2). However, the contact angle for none of the samples was below the threshold value of 65°, at which the required energy expenditures for surface dehydration exceed the energy released from protein molecular adsorption (Vogler, 2012). The lowest contact angle recorded was for the PDO polymeric membrane of 75.7° ± 3.6° reflecting its hydrophilic nature and the capacity of these structure to become saturated, unlike the hydrophobic PCL membranes which tend to retain water molecules at a semi-air and semi-solid interface (Pan et al., 2017; Feng et al., 2020). Upon extended observation (beyond 15–20 s) of a droplet on the PDO surface, it was also noted that the contact angle diminished to 32.1° ± 3.3° underscoring the dynamic nature of hydrophilicity in these materials (Chummun et al., 2018).

It is important to highlight that the hydrophobic nature of the ePTFE polymeric material does not preclude its extensive utilization in the fabrication of stent membranes as well as in the creation of other cardiovascular devices, including vascular prostheses, heart valve prostheses, and patches for heart reconstructions, among others (Roina et al., 2022). This observation suggests that the high hydrophobicity of the materials under study does not negatively impact their suitability for applications within the targeted area. Moreover, it is anticipated that biodegradable polymers will experience structural transformations over time which is expected to result in alterations to their hydrophobic/hydrophilic characteristics, further influencing their interaction with the biological environment.

### 3.3 Mechanical characterization

**FIGURE 3:**
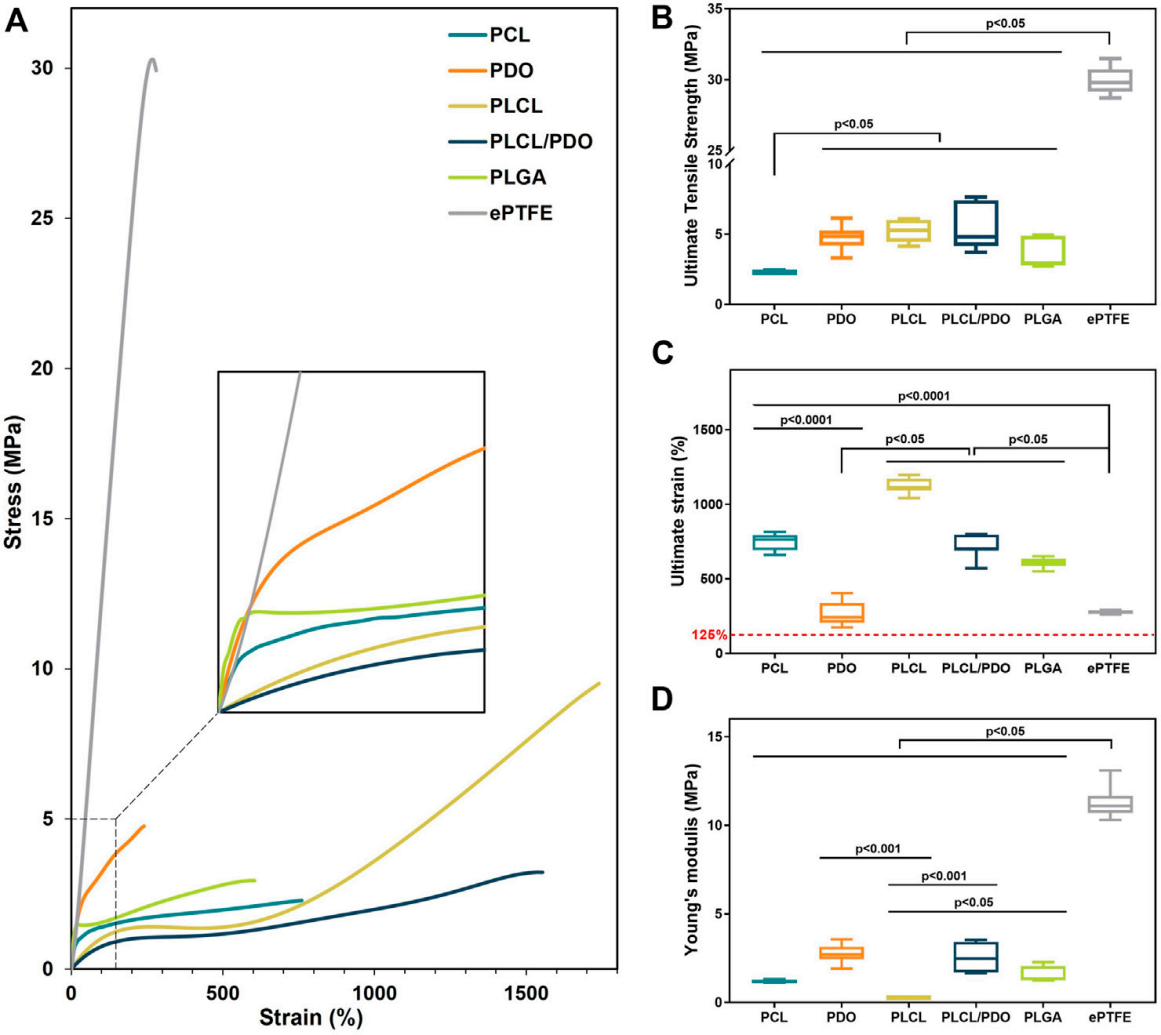
The mechanical properties of electrospun membranes based on PCL, PDO, PLCL, PLCL/PDO, and commercial material ePTFE: Engineering stress-strain curves **(A)**; Ultimate tensile strength **(B)**; Ultimate strain **(C)**; Young’s modulus **(D)**.

Given the necessity for the polymeric membrane covering the stent to withstand a certain degree of load required for the device deployment at the site of a vascular anomaly, the evaluation of the mechanical properties of electrospun materials was deemed crucial (Table 1). Compared to commercial ePTFE, all studied membranes exhibited significantly lower tensile strength, being more than 3.3 times weaker (p < 0.05) (Figures 3A, B). Fibrous materials produced through electrospinning are typically characterized by reduced strength and stiffness in comparison to those created by alternative fabrication methods, such as solution casting (Liao et al., 2011). Nonetheless, the elongation at break for electrospun materials may increase several-fold (Liao et al., 2011).

In this study, the polymeric membrane composed of PCL demonstrated the lowest strength with a value of 2.29 [2.23; 2.36] MPa (p < 0.05). The membranes fabricated from PLGA showed slightly higher resistance to stretching with values of 2.95 [2.77; 4.88] MPa (p < 0.05). Among the electrospun materials, the highest mechanical strengths were observed for PDO, PLCL, and PLCL/PDO samples with respective values of 4.85 [4.20; 5.25], 5.29 [4.45; 6.03], and 4.83 [4.18; 7.41] MPa, without statistically significant differences among them (p > 0.05) (Figures 3A, B). Since the stent-membrane device does not have a predefined requirement for specific strength, this characteristic should not serve as a criterion for exclusion.

Technologically, the process involves the application of the membrane to the stent immediately after its production. The ensuing hybrid stent structure is then affixed to the balloon of the delivery system using a specialized equipment—a crimper—and subsequently expanded to its full diameter at the site of the perforation. From the point of membrane application to the moment of expansion, the diameter of the stent will increase from 1.8 mm to 4 mm, as specified by the manufacturer of the stent prototype employed in this investigation. This translates to a 125% change in the diameter of the membrane marking the minimal elongation that the material must endure without rupture. Furthermore, the elasticity modulus of the membrane emerges as a crucial parameter, given that excessive stiffness in the material could markedly elevate the pressure necessary for stent expansion. The impact of the Young’s modulus on the deployment of the stent is also influenced by the thickness of the coating; a thinner layer covering the stent implies a reduced effect on the deployment process (An et al., 2023).

The ePTFE membranes exhibited the lowest relative elongation at the onset of destruction and the highest Young’s modulus with values of 274.4 [270.4; 282.3]% and 11.10 [10.70; 11.65] MPa, respectively (Figures 3A, C, D). The PDO sample demonstrated a relative elongation comparable to that of ePTFE at 240.7 [205.9; 337.2]% (p = 0.71), though with a modulus of elasticity 4.1 times lower. These findings highlight a limited capacity for material stretching and greater stiffness relative to the other membranes evaluated, potentially restricting PDO’s application as a stent-graft coating. All other matrices significantly exceeded the established boundary of 125% in stretchability (Figure 3C). The lowest value of Young’s modulus, at 0.27 [0.25; 0.28] MPa, was observed for the PLCL polymer (Figure 3D), indicating its greater compliance under stretching with less force applied. The Young’s modulus for the PLCL/PDO composite was significantly higher (p < 0.05) than that for both PCL and PLCL membranes by over 2.1 times. The mechanical properties of the PLCL/PDO material occupied intermediate values between those of PLCL and PDO, a consequence of the dual electrospinning process that merges two distinct processes. The mechanical characteristics, including strength and elastic deformation of PCL and PLGA membranes, showed no significant difference (p > 0.05).

The mechanical properties of fibrous matrices depend significantly on the orientation and thickness of the fibers, adhesion between fibers, and the sliding of one fiber over another, as well as on the chemical nature of the polymer (Rashid et al., 2021). The orientation of molecules within fibers occurs with a reduction in fiber diameter leading to an increase in mechanical strength (Rashid et al., 2021). Therefore, it is expected that with a decrease in fiber diameter, the mechanical properties of electrospun fibers increase. However, despite having the smallest fiber thickness among all the matrices studied, PDO did not exhibit significantly greater strength, and its elasticity was, in contrast, consistently lower compared to the other materials evaluated. Likely, the nature of the polymer plays a key role with individual mechanical property limitations that cannot be overcome by any changes in fiber thickness or concentration, nor by other conditions of electrospinning (Boland et al., 2005).

### 3.4 Testing the deployment of stents covered with polymeric membranes

**FIGURE 4:**
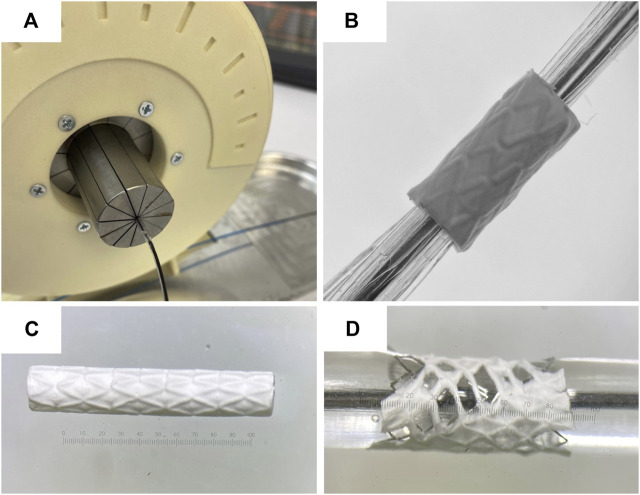
Testing of stent polymer coating: Image of the crimping process of the coated stent onto the balloon using an experimental radial compression equipment **(A)**; stent-membrane sample mounted on the balloon, prior to being expanded to its working diameter **(B)**; stent-membrane with PDO coating immediately after electrospinning and before balloon expansion **(C)**; stent-membrane with PDO coating immediately following balloon expansion **(D)**.

To evaluate the changes in structure and thickness of the polymer coating of the stent-membrane after its expansion with the balloon to a functional state within the human body, the polymer structure before and after expansion was investigated (Figures 4A, B). The diameter of the stent-membrane increased by 125% (from 1.8 mm to 4 mm) upon expansion with the balloon. Although the PDO polymer exhibited satisfactory relative elongation under static uniaxial stretching, the tests revealed destruction of the polymer coating (Figures 4C, D). This occurrence is likely due to uneven expansion of the stent segments leading to the rupture of the polymer matrix in areas subjected to excessive stretching. It is crucial that the polymer coating for balloon-expandable stent membranes includes a “safety margin” and possesses the ability to stretch without sustaining damage. Modifications in the concentration of the PDO solution failed to enhance relative elongation of the samples, a finding supported by literature data (Boland et al., 2005). Consequently, due to its inability to meet these requirements, the PDO polymer was eliminated from subsequent consideration in the study.

**FIGURE 5:**
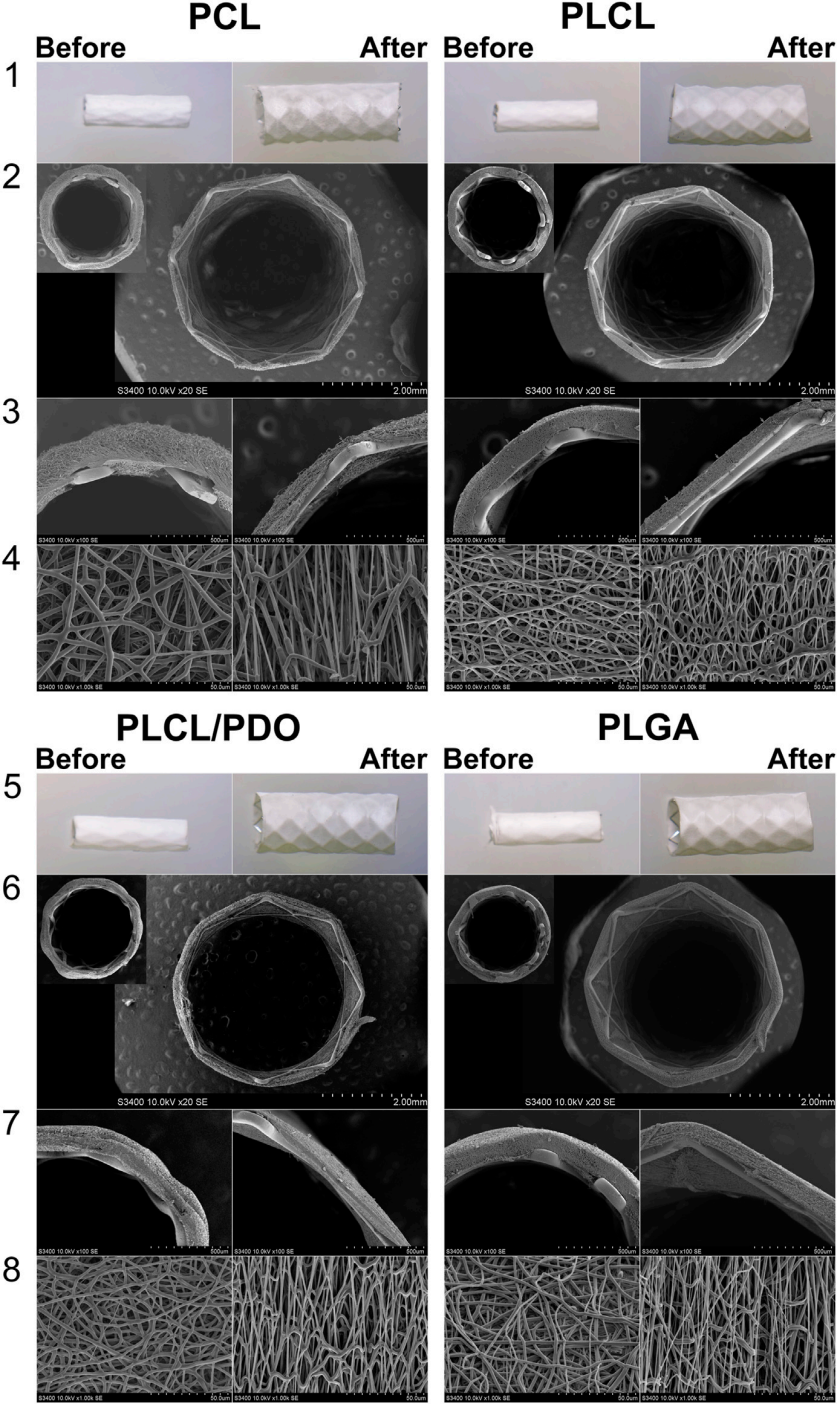
Visualization of stents with biodegradable electrospun membranes made of PCL, PLCL, PLCL/PDO, and PLGA before and after expansion. Images obtained using a stereomicroscope (lines 1, 5) and images obtained by SEM (Scanning Electron Microscopy): cross-sectional view of the stent-membrane, magnification 40 (lines 4, 6); cross-sectional view of the stent-membrane, magnification 100 (lines 3, 7); surface of the stent-membrane visualizing fibers (lines 4, 8).

The coatings of stents with polymers PCL, PLCL, PLCL/PDO, and PLGA exhibited no visible defects, both initially and after expansion with the balloon (Figure 5, lines 1, 5).

To achieve the expansion of a 20 mm long stent coated with PCL, PLCL, PLCL/PDO, and PLGA, pressures of 8, 7, 7, and 10 atm were respectively required, in contrast to the mere 6 atmospheres needed for the expansion of an uncoated stent to its nominal diameter. These findings correlate with the results of the mechanical tests indicating that the Young’s modulus impacts expansion, i.e., the stiffer the material, the greater the required pressure. Across all polymer coatings, fiber elongation accompanied by thinning in the direction of the applied load (circumferentially around the stent) was observed (Figure 5, line 4). However, fibers oriented perpendicular to the load did not exhibit significant changes in thickness. Notably, the coating thickness decreased more substantially opposite the stent struts that is a consequence of their indentation into the polymer layer during balloon manipulation. PCL displayed uneven fiber thinning with certain areas maintaining their original thickness, a phenomenon linked to the polymer structure’s molecular rearrangement under mechanical stress (Figure 5, line 1 for PCL). According to literature, PCL fibers undergo local plastic deformation and narrowing, forming characteristic “necks” in response to viscoelastic stretching which precedes rupture (Delp et al., 2021). The average reduction in PCL fiber thickness was 47% from its initial state with an overall coating thickness reduction of 34% (Table 2).

**TABLE 2:** Fiber diameter and polymer coating thickness before and after expansion of the stent-membrane with the balloon.

Polymer	Fiber diameter (n = 25), µm		Polymer coating thickness (n = 10), µm	
PCL	Before	2.98 [2.65; 3.23]	Before	188.5 [173.3; 198.3]
	After	1.59 [1.40; 2.92]	After	124.0 [108.5; 137.0]
PLCL	Before	1.60 [1.43; 1.69]	Before	159.0 [112.0; 163.0]
	After	1.10 [0.88; 1.25]	After	102.0 [71.9; 117.5]
PLCL/PDO	Before	1.76 [1.57; 1.83]	Before	153.7 [145.3; 165.5]
	After	1.29 [0.99; 1.73]	After	85.0 [56.3; 94.0]
PLGA	Before	1.78 [1.43; 2.04]	Before	126.0 [108.9; 133.7]
	After	1.39 [0.60; 1.77]	After	113.0 [78.1; 121.5]

For the PLCL coating, uniform stretching of the fibers was observed which was different to the segmental narrowing characteristics of PCL. This suggests that the copolymer retains its viscoelastic stretching capabilities under similar load conditions. A reduction in fiber thickness by 31% from its initial measurement and a decrease in overall coating thickness by 35% were observed (Figure 5, line 4, Table 2). Similar to PCL, fibers exhibited thinning in the direction of the applied load (circumferentially around the stent-membrane), while fibers oriented perpendicularly to the load showed no thinning.

After expansion, the thickness of fibers in the PLCL/PDO membrane was reduced by an average of 27% (Figure 5, line 8, Table 2). The corresponding decrease in coating thickness was 44% that was the highest among all the polymeric materials studied (Table 2). A unique aspect of the PLGA membrane was its uneven stretching of unidirectional fibers, potentially attributable to the specific arrangement of fibers during the electrospinning process for this polymer. While fusion of fibers in various directions was observed to some degree in polymeric membranes composed of PCL, PLCL, and PLCL/PDO, this phenomenon was absent in the PLGA membrane. It is therefore posited that during membrane stretching, a series of unattached fibers aligns in the direction of the applied load without altering in thickness, whereas other fibers undergo stretching. On average, the thickness of fibers decreased by 22%, with the coating thickness itself only reducing by 10% (Figure 5, line 4, Table 2). Fusion among fibers might result from a significant presence of solvent vapor within the system (Li et al., 2017). When comparing the electrospinning process of PLGA to that of other polymers, a higher solution concentration (30%) was noted leading to a diminished solvent presence, which may have impacted these findings.

Thus, the stretching of membranes based on PCL, PLCL, PLCL/PDO, and PLGA formed directly on the stent, within the specified range of elongation did not lead to fiber rupture or disruption of the materials structure. This process was accompanied by fiber thinning and a reduction in the distance between them, consequently leading to a decrease in the thickness of the polymer coating which should be considered in the design of the stent-membrane. The commercially available PK Papyrus stent, which currently shows positive results in addressing coronary artery perforations, is featured with a membrane thickness of about 90 μm at full expansion (Kandzari and Birkemeyer, 2019), comparable to our results. The PK Papyrus stent can expand to a maximum diameter of 3.50 mm for stents initially measuring 2.5 and 3.0 mm in diameter (Kandzari and Birkemeyer, 2019). In contrast, the initial diameter of our device is significantly smaller (1.8 mm), offering enhanced versatility.

### 3.5 Cytocompatibility assessment

**FIGURE 6:**
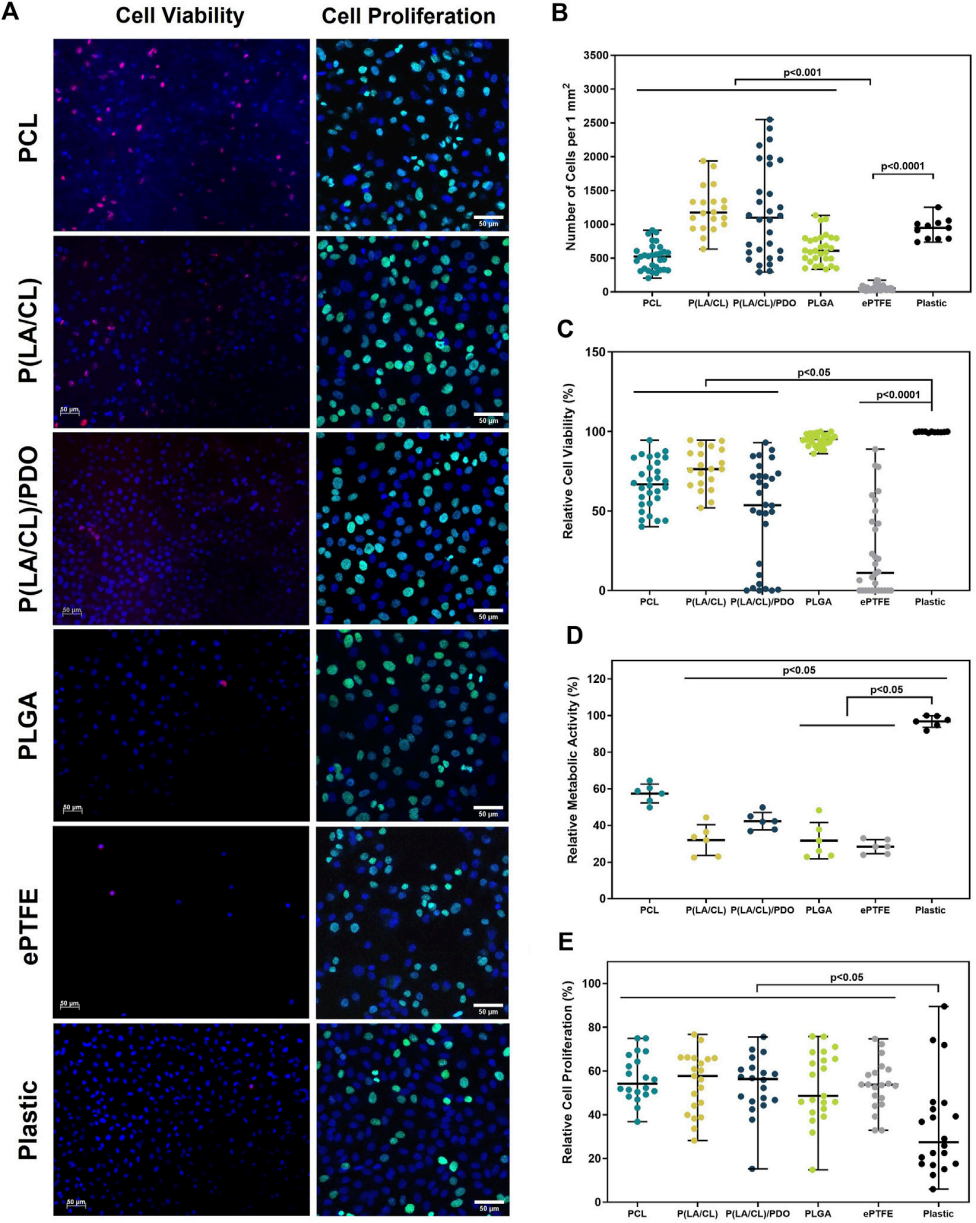
Cytotoxicity assessment of polymer membranes PCL, PLCL, PLCL/PDO, PLGA, and ePTFE. Representative microphotographs obtained from the fluorescent staining of Ea. hy 926 cells cultured on polymeric materials and culture plastic **(A)**. The total number of cells adhered to the surface of polymeric membranes **(B)**. The relative number of viable cells on the surface of polymeric membranes **(C)**. The relative metabolic activity of the cells **(D)**. The relative number of proliferating on the surface of polymeric membranes cells **(E)**.

To assess the cytotoxicity of the developed polymeric materials, endothelial Ea. hy926 cells were cultured on the surfaces of the obtained membranes. The ability of the materials to support adhesion and viability of the endothelial cells adhered to the surface were evaluated in a comparative aspect. Based on the level of viability, the studied polymers were divided into three categories: with relatively high (>75%), medium (75% > × >50%), and low (<15%) viability (Figure 6).

The PLGA membranes demonstrated high cell viability, 95.4 [91.1; 97.7]%, which was comparable to the culture plastic at 99.8 [99.6; 100.0]% (p > 0.9999). The samples of PLCL, PCL, and PLCL/PDO showed a medium level of cell viability equal to 76.4 [66.5; 88.1]%, 66.8 [54.7; 81.3]%, and 53.7 [8.4; 72.5]% respectively. The viability on these materials was on average 25% lower compared to the control on plastic and with PLGA samples (p < 0.05). The surface of ePTFE material was found to support a significantly reduced level of endothelial cell viability – 11.2 [0.0; 45.0]% which was statistically different from all other groups in the study (p < 0.05) (Figures 6A, C). This outcome is attributed primarily to diminished capacity for cellular adhesion of the ePTFE samples (Figures 6A, B) which is characteristic of the material high hydrophobicity and the chemical properties of its functional groups (Lu et al., 2013).

Studying the metabolic activity of cells allowed for the assessment of cytotoxicity in the respective well of the sample without considering the number of cells (Figure 6D). Samples of ePTFE, PLGA, and PLCL demonstrated lower metabolic activity compared to culture plastic (p < 0.05). On PCL, the indicators of cell metabolic activity were higher compared to ePTFE, PLGA, and PLCL, yet they did not significantly differ from the control samples and PLCL/PDO (p > 0.05).

In the context of promoting the proliferative activity of cells attached to the membrane surfaces, samples composed of PLGA, PLCL, PLCL/PDO, and PCL demonstrated optimal outcomes characterized by elevated cell proliferation rates (Figures 6A, E). For ePTFE samples, while the cell colonization density was lower compared to other membrane types, notable levels of proliferative activity highlighted these matrices’ proficiency in supporting the viability of adhered cells. When evaluating the outcomes of cell proliferative activity, it is crucial to acknowledge that vigorous cell division resulting in dense surface colonization may initiate contact inhibition, thus curbing unchecked cell proliferation. Consequently, a high cell density might coexist with comparatively reduced proliferative activity. This phenomenon clarifies why the control samples exhibit high metabolic activity and cell density while showing relatively lower proliferative activity compared to certain materials (Figures 6A, E).

The results of the cell culturing indicate the biocompatibility and minimal cytotoxicity of the studied materials. Despite the low adhesive capability, ePTFE samples demonstrated satisfactory metabolic and proliferative activities, with the electrospun samples being comparable to ePTFE in terms of biocompatibility parameters. Considering the widespread use of ePTFE in medicine, including for coronary stent coatings (Roina et al., 2022), the potential applications of PCL, PLCL, PLCL/PDO, and PLGA polymers for stent-membrane development are evident. Additionally, the capacity of these materials to facilitate the adhesion and proliferation of endothelial cells suggests a potential for endothelialization of the membranes inner surface. By isolating the synthetic material from contact with blood, this could ultimately reduce the risk of thrombosis and inflammatory complications. The literature also confirms the greater ability of electrospun matrices based on PCL and combinations with PCL for endothelialization compared to ePTFE (Pfeiffer et al., 2014).

### 3.6 Platelet adhesion

**FIGURE 7:**
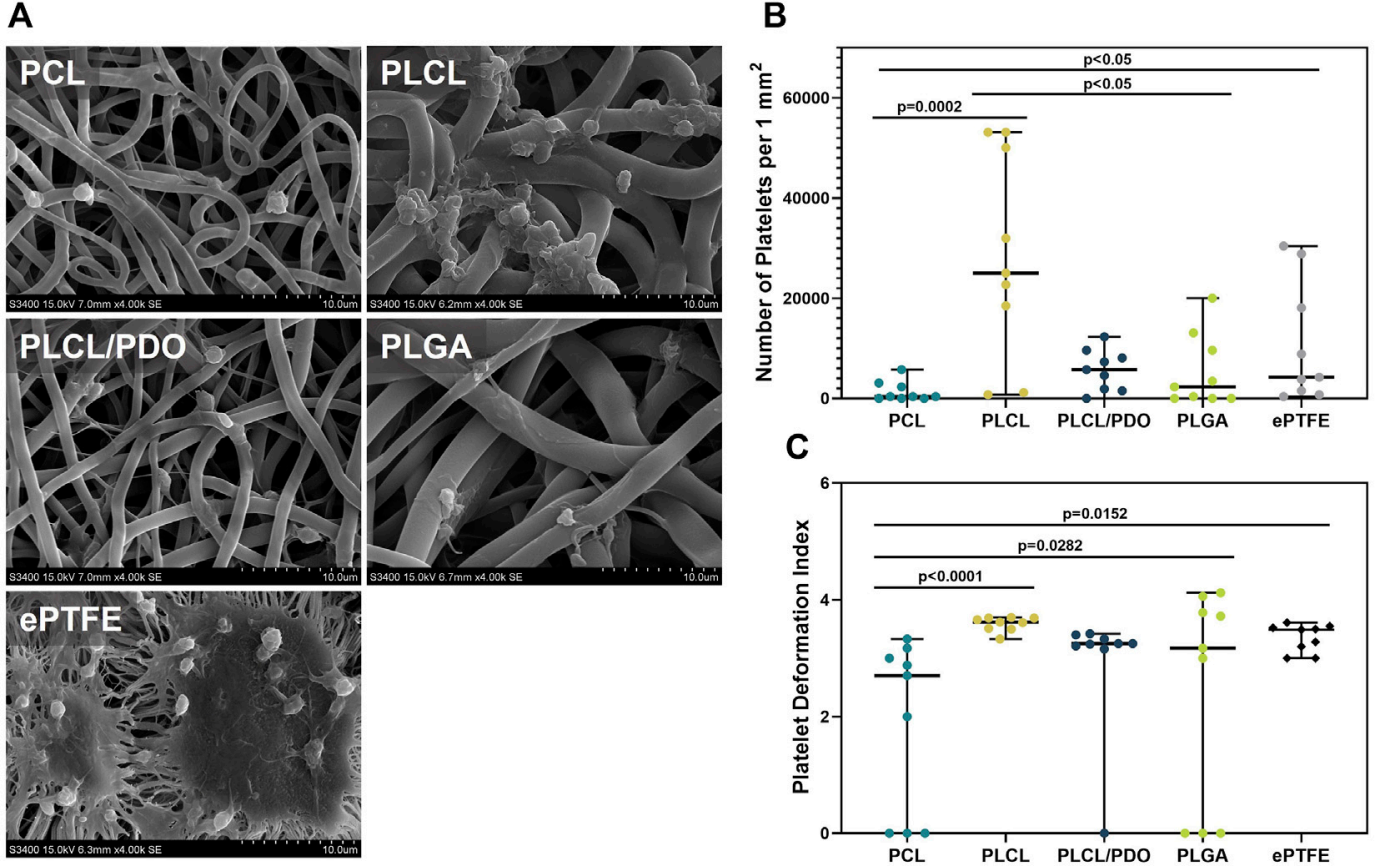
Platelet adhesion on polymer membranes PCL, PLCL, PLCL/PDO, PLGA, and ePTFE. Representative SEM images of platelet adhesion, magnification 4,000 **(A)**. The number of platelets adhered to the surface of polymeric membranes **(B)**. The deformation index of platelets adhered to the membrane surface **(C)**.

Since the stent-membrane being developed will be located at a site of constant contact with blood, it is particularly important to assess the polymer coating’s potential to thrombus formation. For this purpose, the polymers interaction with platelet-rich plasma was studied. Adhesion of platelets was observed to some extent across all polymeric materials (Figure 7A). Differences in the number and degree of platelet activation were found between the samples used in the experiment.

The ePTFE showed a high degree of platelet adhesion: the number of formed elements detected on the surface of the samples amounted to 4,238.0 per 1 mm^2^, primarily featuring platelets of III and IV types of deformation (Figures 7A–C). Regarding the membranes produced by electrospinning, the minimum values were obtained for PCL and the maximum for PLCL which was 11 times less (p = 0.002) and 5.9 times more (p = 0.081) than that of ePTFE samples, respectively (Figures 7A, B). The high thrombogenicity of the PLCL material may be associated with the presence of polylactide segments in the polymer structure, which according to literature, have a high affinity for platelet adhesion (Yakub et al., 2016).

On the surface of PLCL, type IV platelets predominated, while type III platelets were characteristic for PLCL/PDO and PCL. PLGA indicated the presence of types III, IV, and V platelets in equal proportions (Figures 7A, C). Another advantage of PCL was the absence of type V platelets and a significantly lower deformation index relative to other polymer materials (p < 0.05). Among all studied polymeric membranes, only the PLCL sample was significantly more thrombogenic (p < 0.05) compared to the ePTFE material. Consequently, this type of polymer was excluded from further experiments assessing hemocompatibility *in vivo*.

The thrombogenicity of the ePTFE polymer is attributed both to its high hydrophobicity and the chemical nature of its functional groups. This characteristic is a limiting factor for the use of the biomaterial in certain applications, specifically, unmodified ePTFE is unsuitable for replacing small-diameter vessels (Kim et al., 2019). Meanwhile, PCL and its combinations with other biodegradable biocompatible polymers, including PDO, find applications in the development of tissue-engineered prostheses for such vessels (Pan et al., 2017). Confirming our findings, PCL demonstrated satisfactory hemocompatibility in *in vivo* experiments (Pan et al., 2017).

### 3.7 Hemocompatibility and biodegradation *in vivo*

In contrast to materials like polyurethane or ePTFE, which are utilized in commercial stents, the polymers we selected possess the capability for complete biodegradation, potentially minimizing the risks associated with long-term inflammatory and thrombotic complications. Thus, a crucial criterion for evaluating material suitability extended beyond hemocompatibility to include the biodegradation behavior *in vivo*. Samples of polymer material were implanted into the abdominal aorta of rats, with ePTFE serving as a benchmark for assessing inflammatory response and thrombogenic potential.

The comprehensive testing protocol, which included implantation periods of 5 and 20 days, was successfully completed by three of the four polymers under study: PCL, PLCL/PDO, and ePTFE. All laboratory animals implanted with PLGA patches died within the first day following the procedure. Necropsy of these animals revealed internal hemorrhaging into the abdominal cavity and perforations in the polymeric membranes. The ruptures in the PLGA membrane are likely attributed to the rapid material degradation within the physiological conditions of the animal, coupled with mechanical stress from blood pressure (Duan et al., 2007). The biodegradation rate of PLGA with a 50/50 ratio is 1–2 weeks (Gentile et al., 2014), which is significantly faster than the degradation rate of PCL and PLA electrospun matrices, which can take up to 2 years for complete dissolution within the organism (Porjazoska-Kujundziski and Chamovska, 2017). Furthermore, literature data confirm that despite the minimal mass loss of the PLGA polymer, destruction of the amorphous sections of the polymer fibers occurs already at the initial stages of degradation, leading to a substantial reduction in the mechanical properties of the materials (Miao et al., 2021).

**FIGURE 8:**
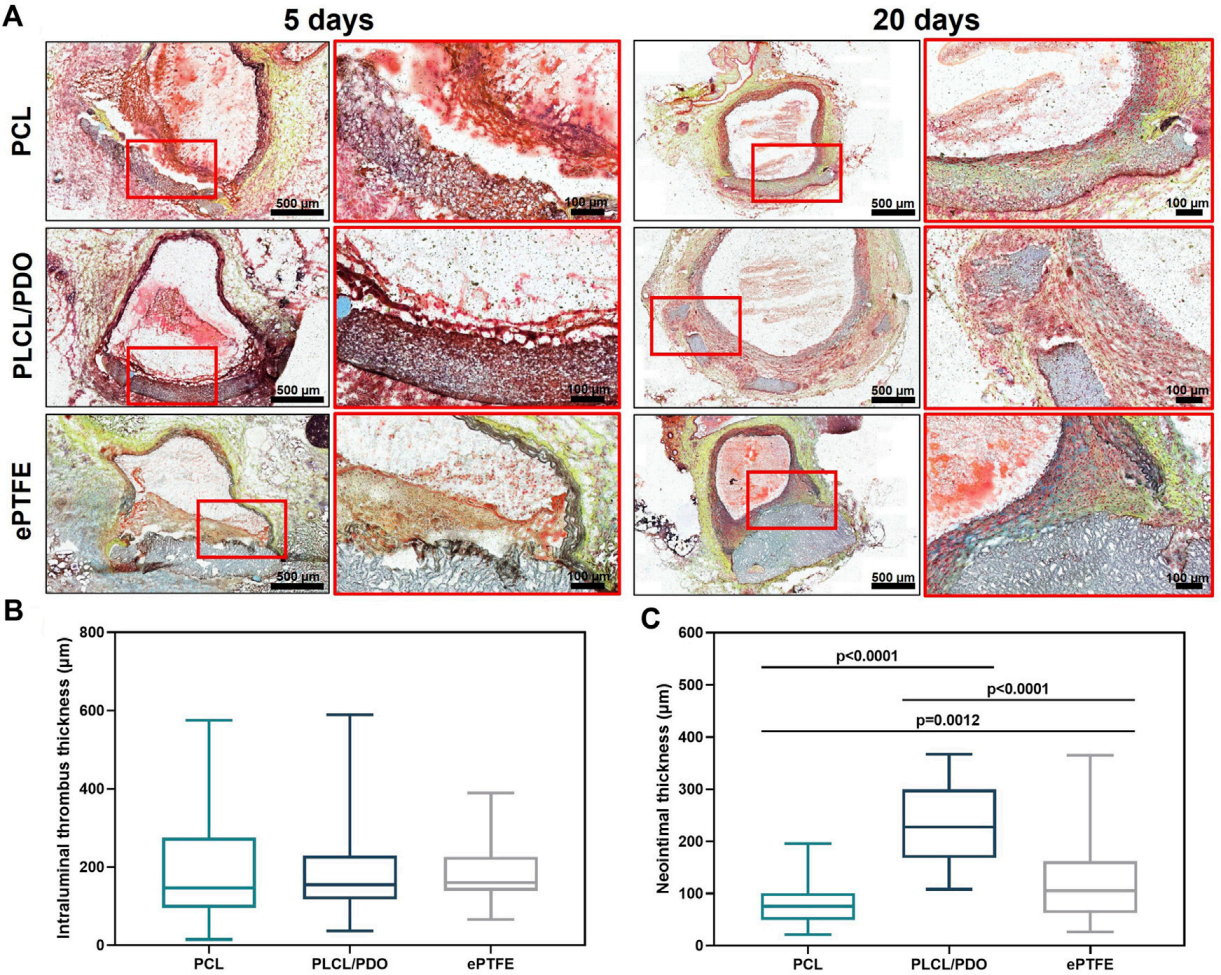
Cross-sections of rat aorta removed 5 and 20 days after implantation of the studied polymeric membranes PCL, PLCL/PDO, and ePTFE (Russell-Movat pentachrome staining) **(A)**. Thrombus thickness after 5 days of implantation **(B)**. Neointima thickness after 20 days of implantation **(C)**.

The reaction of surrounding tissues to the implantation of polymer membranes made of PCL, PLCL/PDO, and ePTFE after 5 days did not significantly differ among the samples. Thus, all polymer patches were covered with loose thrombotic masses, which included significant accumulations of segmented neutrophils (Figure 8A). There were no significant differences (p > 0.8) in the thickness of the thrombotic masses formed on the surface of the studied polymer patches (Figure 8B). In all cases, the thrombotic masses did not significantly affect the patency of the aorta. Also, there were no signs of biodegradation of any of the studied polymers.

Twenty days post-implantation, we observed regeneration of the vascular wall with the formation of neointima over the polymer membranes (Figures 8A, C). The structure of the latter markedly differed from that of the native vessel wall by the absence of elastic fibers. No signs of thrombosis were noted.

Control samples made of ePTFE had clear contours and bore no signs of biodegradation, although we observed the penetration of macrophages into pores within the polymer left by the surgical needle during the sewing of the membrane to the aorta wall (Figure 8A). In contrast, membranes made from PLCL/PDO underwent fragmentation, with individual polymer fragments retaining clear contours (Figure 8A). The biodegradation of PLCL/PDO was accompanied by the most intense regeneration of the vascular wall with the formation of the thickest neointima compared to ePTFE and PCL samples (p < 0.0001; Figure 8C). Finally, PCL membranes were thinned, had barely visible outlines, and were uniformly infiltrated by cells (Figure 8A). The neointima formed on their surface had the minimal thickness compared to other polymer samples (Figure 8C).

**FIGURE 9:**
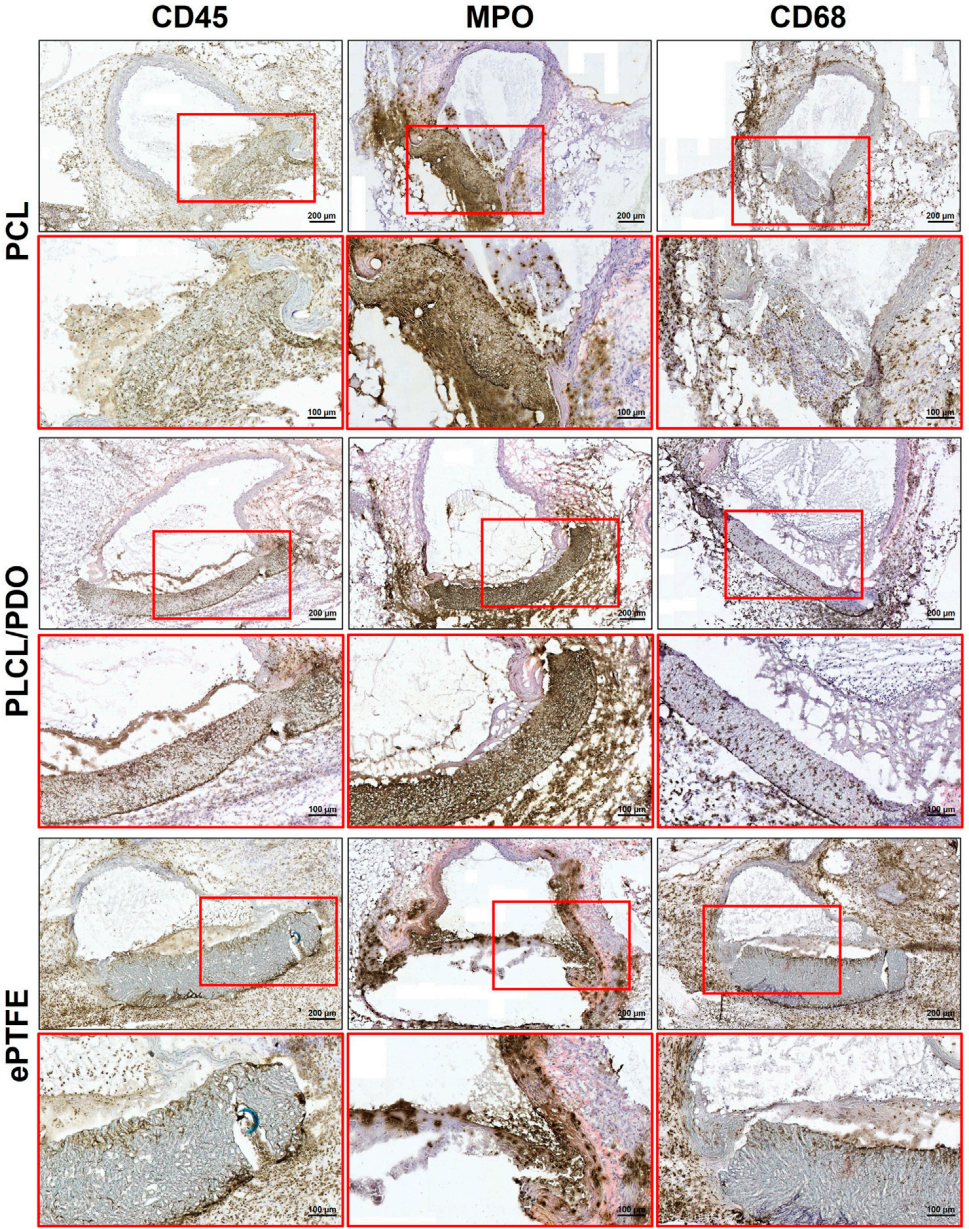
Immunohistochemical staining of cross-sections of rat aorta removed 5 days after implantation of the studied polymeric membranes PCL, PLCL/PDO and ePTFE.

Immunohistochemical staining of samples explanted after 5 days demonstrated an acute inflammatory response to all types of polymeric membranes (Figure 9). The inflammation was characterized by aggressive neutrophil infiltration of the peri-implantation space (MPO+). Furthermore, much of the perivascular adipose tissue adjacent to the aorta and polymeric patches was abundant with scattered macrophages (CD68^+^), indicating activation of the innate immune system, likely due to damage to the aorta and adjacent tissues as a result of the surgical intervention.

**FIGURE 10:**
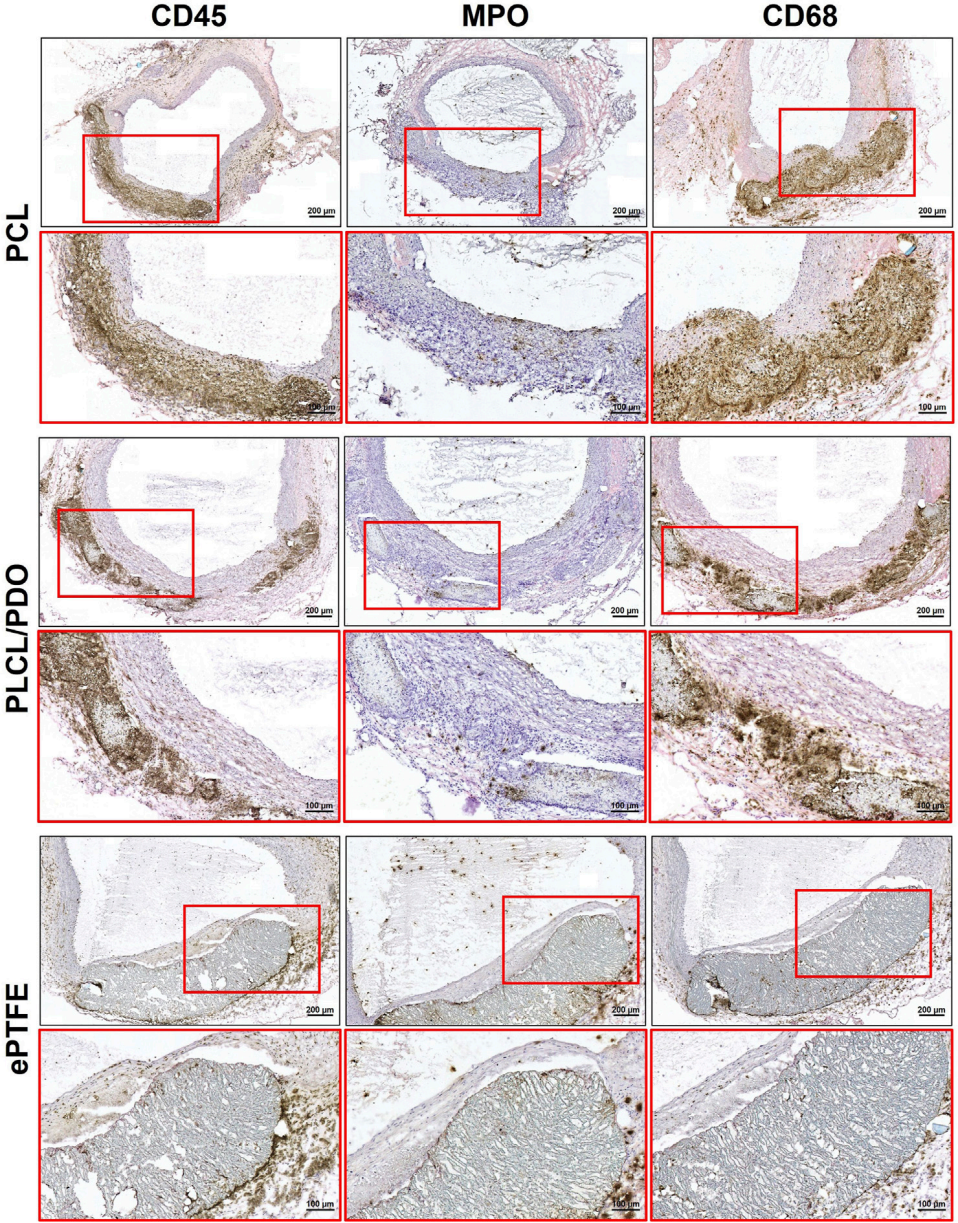
Immunohistochemical staining of rat aorta cross-sections removed 20 days after implantation of the studied polymeric membranes PCL, PLCL/PDO and ePTFE.

In turn, 20 days post-implantation, there was a minimal presence of neutrophils (MPO+) while intense macrophage infiltration (CD68^+^) persisted. It is important to emphasize that dense accumulations of macrophages were located exclusively near the polymer samples and their fragments, whereas the perivascular tissue surrounding the aorta was largely free from immune cells. These findings indicate a transition of the inflammatory response from the acute to the chronic phase (Figure 10).

The results of *in vivo* testing demonstrate that the implantation of PCL, PLCL/PDO, and ePTFE into the rat’s abdominal aorta at early stages induces an identical thrombo-inflammatory response from the surrounding tissues. This indicates that PLCL/PDO and PCL have biocompatible properties similar to those of the ePTFE material used in modern surgery.

At the same time, unlike ePTFE, PCL and PLCL/PDO membranes are subject to biodegradation when functioning in the body of a rat over a long period, occurring through different mechanisms. Thus, PCL membranes show a tendency for gradual destruction and replacement by a connective tissue due to infiltration by recipient cells, whereas PLCL/PDO samples fragment. The observed differences in biodegradation between PLCL/PDO and PCL can be explained by the different pore diameters: PCL is characterized by large pores, which may facilitate easy penetration and even distribution of immune cells within the polymer, while PLCL/PDO features a fine-porous structure, and cell infiltration of this material is possible only in certain areas. Moreover, the PLCL/PDO membrane, being a composite material, exhibits variable resistance to biodegradation across different areas due to the uneven distribution of its components, leading to a focal pattern of degradation. The rate of biodegradation of PDO exceeds that of PCL, which could be the cause of the effect we observed (Zhou et al., 2019; Dias et al., 2022). It is known that the degradation rate of PCL and PLA is approximately 2 years, whereas the degradation rate of PDO is only 30 weeks (Porjazoska-Kujundziski and Chamovska, 2017). The *in vivo* biodegradation model in rats does not directly correlate with the degradation rate in the human body; moreover, factors such as porosity, implantation site, and material thickness can significantly influence degradation rates. However, in a comparative aspect, we observed a reduction in degradation times for PLCL/PDO materials. Nevertheless, this should be sufficient to fulfill the closure functions in the therapy of coronary artery perforations.

### 3.8 Permeability assessment

The results indicate that expanded polytetrafluoroethylene (ePTFE) demonstrated almost negligible water permeability, with values below the detection threshold both in its initial state and under 125% deformation (Table 3). This near absolute impermeability is consistent with its highly crystalline structure and tight fiber weave, which effectively prevent water molecules from passing through. Previous studies have also highlighted PTFE’s low permeability, aligning with the current findings (Luo et al., 2021).

**TABLE 3:** Water permeability of polymer membranes before (0%) and after stretching by 125%.

	0%		125%	
Polymer	Leakage volume, mL	Permeability, mL/min/mm^2^	Leakage volume, mL	Permeability, mL/min/mm^2^
PCL	389 ± 110	0.78 ± 0.22	405 ± 0.63	0.81 ± 0.13
PDO	320 ± 84	0.64 ± 0.17	356 ± 64	0.71 ± 0.13
PLCL	7 ± 2	0.014 ± 0.004	6 ± 3	0.012 ± 0.006
PLCL/PDO	2 ± 1	0.004 ± 0.002	2 ± 1	0.004 ± 0.002
PLGA	4 ± 2	0.008 ± 0.004	102 ± 13	0.20 ± 0.03
ePTFE	<1	<0.002	<1	<0.002

PLCL and the PLCL/PDO composite exhibited the best results in terms of permeability (below 0.02 mL/min/mm^2^), indicating their potential suitability for coronary stent graft applications. This superior performance can be attributed to the copolymer structure of PLCL, which balances the flexibility of caprolactone with the mechanical strength of lactide. PLGA showed good initial water permeability properties; however, its performance deteriorated significantly upon deformation to 125% (Table 3). This decline can be attributed to the disruption of the polymer matrix under mechanical stress, leading to the formation of microcracks or voids that facilitate water passage.

PDO demonstrated substantial leakage in its initial state and ruptured under 125% deformation (Table 3). The high initial leakage volume suggests that PDO’s hydrophilic nature contributes to greater water absorption and passage through the polymer matrix.

PCL exhibited the highest leakage volume and permeability among the tested polymers, both in its initial state and after 125% deformation, which may limit its applicability for coronary stent graft applications (Table 3). The high permeability observed suggests that PCL has a more open polymer matrix structure, allowing water molecules to pass through more readily. Additionally, deformation may exacerbate these issues by further disrupting the polymer network and creating additional pathways for water leakage.

However, it is important to note that blood viscosity is significantly higher than that of water used in the experiment, and proteins and blood components instantly occlude pores upon contact with the membrane, thereby altering its performance. This explains why the *in vivo* test for PCL was successful. Nonetheless, a comprehensive *in vivo* test involving perforation is necessary to reach a definitive conclusion.

## 4 Conclusion

This paper has described a comparative study on bioresorbable polymer materials for coating coronary artery stents. The results of the conducted functional tests have demonstrated that electrospinning is a convenient method to cover stents with membranes promising for the treatment of coronary artery perforations. The data obtained from mechanical testing, permeability, bio- and hemocompatibility assessments, and the dynamics of biodegradation have indicated that the membranes produced from PLCL/PDO would be advantageous for further research. Our findings can serve as the basis for preparing a preclinical study of the stent-structure in large laboratory animals. It is important to mention that practical implementation of the materials of interest requires their performance to be characterized under conditions as close as possible to real-life biomedical applications.

## Data Availability

The original contributions presented in the study are included in the article/supplementary material, further inquiries can be directed to the corresponding author.

## References

[B1] Abubakar M. Javed I. Rasool H. F. Raza S. Basavaraju D. Abdullah R. M. (2023). Advancements in percutaneous coronary intervention techniques: a comprehensive literature review of mixed studies and practice guidelines. Cureus 15, e41311. 10.7759/cureus.41311 37539426 PMC10395399

[B2] Agathos E. A. Tomos P. I. Kostomitsopoulos N. Koutsoukos P. G. (2019). Calcitonin as an anticalcification treatment for implantable biological tissues. J. Cardiol. 73, 179–182. 10.1016/j.jjcc.2018.07.010 30377016

[B3] Al-Mukhaini M. Panduranga P. Sulaiman K. Riyami A. A. Deeb M. Riyami M. B. (2011). Coronary perforation and covered stents: an update and review. Heart Views 12, 63–70. 10.4103/1995-705X.86017 22121463 PMC3221194

[B4] An W. Ye J. Han B. Wang X. Han C. Gao J. (2023). Efficacy and safety of self-made covered coronary stent in the treatment of coronary artery perforation. BMC Cardiovasc. Disord. 23, 537. 10.1186/s12872-023-03575-3 37923982 PMC10625290

[B5] Avula V. Karacsonyi J. Kostantinis S. Simsek B. Rangan B. V. Gutierrez A. A. (2022). Incidence, treatment, and outcomes of coronary artery perforation during percutaneous coronary intervention. J. Invasive Cardiol. 34, E499–E504. 10.25270/jic/21.00358 35714223

[B6] Boland E. D. Coleman B. D. Barnes C. P. Simpson D. G. Wnek G. E. Bowlin G. L. (2005). Electrospinning polydioxanone for biomedical applications. Acta Biomater. 1, 115–123. 10.1016/j.actbio.2004.09.003 16701785

[B7] Boodagh P. Guo D. J. Nagiah N. Tan W. (2016). Evaluation of electrospun PLLA/PEGDMA polymer coatings for vascular stent material. J. Biomater. Sci. Polym. Ed. 27, 1086–1099. 10.1080/09205063.2016.1176715 27137629

[B8] Briguori C. Nishida T. Anzuini A. Di Mario C. Grube E. Colombo A. (2000). Emergency polytetrafluoroethylene-covered stent implantation to treat coronary ruptures. Circulation 102, 3028–3031. 10.1161/01.CIR.102.25.3028 11120690

[B9] Chausse V. Casanova-Batlle E. Canal C. Ginebra M.-P. Ciurana J. Pegueroles M. (2023). Solvent-cast direct-writing and electrospinning as a dual fabrication strategy for drug-eluting polymeric bioresorbable stents. Addit. Manuf. 71, 103568. 10.1016/j.addma.2023.103568

[B10] Chen S. Lotan C. Jaffe R. Rubinshtein R. Ben-Assa E. Roguin A. (2015). Pericardial covered stent for coronary perforations. Catheter. Cardiovasc. Interv. 86, 400–404. 10.1002/ccd.26011 26155775

[B11] Chummun I. Bhaw-Luximon A. Jhurry D. (2018). Modulating matrix-multicellular response using polysucrose-blended with poly-L-lactide or polydioxanone in electrospun scaffolds for skin tissue regeneration. J. Biomed. Mater. Res. A 106, 3275–3291. 10.1002/jbm.a.36527 30367544

[B12] Delp A. Becker A. Hülsbusch D. Scholz R. Müller M. Glasmacher B. (2021). *In situ* characterization of polycaprolactone fiber response to quasi-static tensile loading in scanning electron microscopy. Polym. (Basel) 13, 2090. 10.3390/polym13132090 PMC8271998 34202874

[B13] Dias J. R. Sousa A. Augusto A. Bártolo P. J. Granja P. L. (2022). Electrospun polycaprolactone (PCL) degradation: an *in vitro* and *in vivo* study. Polymers 14, 3397. 10.3390/polym14163397 36015652 PMC9415937

[B14] Duan B. Wu L. Yuan X. Hu Z. Li X. Zhang Y. (2007). Hybrid nanofibrous membranes of PLGA/chitosan fabricated via an electrospinning array. J. Biomed. Mater. Res. A 83A, 868–878. 10.1002/jbm.a.31408 17567858

[B15] Feng B. Ji T. Wang T. Fu W. Ye L. Zhang H. (2020). Engineering cartilage tissue based on cartilage-derived extracellular matrix cECM/PCL hybrid nanofibrous scaffold. Mater. and Des. 193, 108773. 10.1016/j.matdes.2020.108773

[B16] Gentile P. Chiono V. Carmagnola I. Hatton P. V. (2014). An overview of poly(lactic-co-glycolic) acid (PLGA)-based biomaterials for bone tissue engineering. Int. J. Mol. Sci. 15, 3640–3659. 10.3390/ijms15033640 24590126 PMC3975359

[B17] Gruberg L. Pinnow E. Flood R. Bonnet Y. Tebeica M. Waksman R. (2000). Incidence, management, and outcome of coronary artery perforation during percutaneous coronary intervention. Am. J. Cardiol. 86, 680–682. 10.1016/s0002-9149(00)01053-5 10980224

[B18] Han J. Lazarovici P. Pomerantz C. Chen X. Wei Y. Lelkes P. I. (2011). Co-electrospun blends of PLGA, gelatin, and elastin as potential nonthrombogenic scaffolds for vascular tissue engineering. Biomacromolecules 12, 399–408. 10.1021/bm101149r 21182235

[B19] Hu J. J. Chao W. C. Lee P. Y. Huang C. H. (2012). Construction and characterization of an electrospun tubular scaffold for small-diameter tissue-engineered vascular grafts: a scaffold membrane approach. J. Mech. Behav. Biomed. Mater. 13, 140–155. 10.1016/j.jmbbm.2012.04.013 22854316

[B20] Iqbal J. Gunn J. Serruys P. W. (2013). Coronary stents: historical development, current status and future directions. Br. Med. Bull. 106, 193–211. 10.1093/bmb/ldt009 23532779

[B21] Kandzari D. E. Birkemeyer R. (2019). PK Papyrus covered stent: device description and early experience for the treatment of coronary artery perforations. Catheter. Cardiovasc. Interv. 94, 564–568. 10.1002/ccd.28306 31033148

[B22] Kim D. Chung J. J. Jung Y. Kim S. H. (2019). The effect of Substance P/Heparin conjugated PLCL polymer coating of bioinert ePTFE vascular grafts on the recruitment of both ECs and SMCs for accelerated regeneration. Sci. Rep. 9, 17083. 10.1038/s41598-019-53514-6 31745143 PMC6863833

[B23] King W. E. Bowlin G. L. (2022). Near-field electrospinning of polydioxanone small diameter vascular graft scaffolds. J. Mech. Behav. Biomed. Mater. 130, 105207. 10.1016/j.jmbbm.2022.105207 35367688

[B24] Kufner S. Schacher N. Ferenc M. Schlundt C. Hoppmann P. Abdel-Wahab M. (2019). Outcome after new generation single-layer polytetrafluoroethylene-covered stent implantation for the treatment of coronary artery perforation. Cardiovasc. Inerv. 93, 912–920. 10.1002/ccd.27979 30467994

[B25] Kuno T. Ohata T. Nakamaru R. Sawano M. Kodaira M. Numasawa Y. (2023). Long-term outcomes of periprocedural coronary dissection and perforation for patients undergoing percutaneous coronary intervention in a Japanese multicenter registry. Sci. Rep. 13, 20318. 10.1038/s41598-023-47444-7 37985895 PMC10662469

[B26] Kuznetsov K. A. Murashov I. S. Chernonosova V. S. Chelobanov B. P. Stepanova A. O. Sergeevichev D. S. (2020). Vascular stents coated with electrospun drug-eluting material: functioning in rabbit iliac artery. Polymers. 12, 1741. 10.3390/polym12081741 32759856 PMC7465440

[B27] Kwok O. H. Ng W. Chow W. H. (2001). Late stent thrombosis after successful rescue of a major coronary artery rupture with a polytetrafluoroethylene-covered stent. J. Invasive Cardiol. 13, 391–394. 11385155

[B28] Lemmert M. E. van Bommel R. J. Diletti R. Wilschut J. M. de Jaegere P. P. Zijlstra F. (2017). Clinical characteristics and management of coronary artery perforations: a single-center 11-year experience and practical overview. J. Am. Heart Assoc. 6, e007049. 10.1161/JAHA.117.007049 28939719 PMC5634316

[B29] Li H. Zhu C. Xue J. Ke Q. Xia Y. (2017). Enhancing the mechanical properties of electrospun nanofiber mats through controllable welding at the cross points. Macromol. Rapid Commun. 38, 1600723. 10.1002/marc.201600723 PMC5532542 28295875

[B30] Liao G. Y. Chen L. Zeng X. Y. Zhou X. P. Xie X. L. Peng E. J. (2011). Electrospun poly(L-lactide)/poly(ε-caprolactone) blend fibers and their cellular response to adipose-derived stem cells. J. Appl. Polym. Sci. 120, 2154–2165. 10.1002/app.33398

[B31] Lu S. Zhang P. Sun X. Gong F. Yang S. Shen L. (2013). Synthetic ePTFE grafts coated with an anti-cd133 antibody-functionalized heparin/collagen multilayer with rapid *in vivo* endothelialization properties. ACS Appl. Mater. Interfaces 5, 7360–7369. 10.1021/am401706w 23859593

[B32] Luo Y. Gong X. S. Xu Z. Meng K. Zhang K.-Q. Zhao H. (2021). PTFE electrospun stent graft—preparation, properties and its industrialization prospect. Chem. Res. Chin. Univ. 37, 589–597. 10.1007/s40242-021-1177-4

[B33] Miao Y. Cui H. Dong Z. Ouyang Y. Li Y. Huang Q. (2021). Structural evolution of polyglycolide and poly(glycolide-co-lactide) fibers during *in vitro* degradation with different heat-setting temperatures. ACS Omega 6, 29254–29266. 10.1021/acsomega.1c04974 34746613 PMC8567347

[B34] Pan Y. Zhou X. Wei Y. Zhang Q. Wang T. Zhu M. (2017). Small-diameter hybrid vascular grafts composed of polycaprolactone and polydioxanone fibers. Sci. Rep. 7, 3615. 10.1038/s41598-017-03851-1 28620160 PMC5472623

[B35] Pfeiffer D. Stefanitsch C. Wankhammer K. Müller M. Dreyer L. Krolitzki B. (2014). Endothelialization of electrospun polycaprolactone (PCL) small caliber vascular grafts spun from different polymer blends. J. Biomed. Mater. Res. A 102, 4500–4509. 10.1002/jbm.a.35123 24532056

[B36] Porjazoska-Kujundziski A. Chamovska D. (2017). Biodegradable polymers suitable for tissue engineering and drug delivery systems. Zastita Mater. 58 (3), 333–348. 10.5937/ZasMat1703333P

[B37] Rashid T. U. Gorga R. E. Krause W. E. (2021). Mechanical properties of electrospun fibers—a critical review. Adv. Eng. Mater. 23, 2100153. 10.1002/adem.202100153

[B38] Rickel A. P. Deng X. Engebretson D. Hong Z. (2021). Electrospun nanofiber scaffold for vascular tissue engineering. Mater. Sci. Eng. C Mater. Biol. Appl. 129, 112373. 10.1016/j.msec.2021.112373 34579892 PMC8486306

[B39] Roina Y. Auber F. Hocquet D. Herlem G. (2022). ePTFE-based biomedical devices: an overview of surgical efficiency. J. Biomed. Mater. Res. B Appl. Biomater. 110, 302–320. 10.1002/jbm.b.34928 34520627

[B40] Secco G. G. Serdoz R. Kilic I. D. Caiazzo G. Mattesini A. Parisi R. (2016). Indications and immediate and long-term results of a novel pericardium covered stent graft: consecutive 5 year single center experience. Catheter. Cardiovasc. Interv. 87, 712–719. 10.1002/ccd.26131 26541909

[B41] Vogler E. A. (2012). Protein adsorption in three dimensions. Biomaterials 33, 1201–1237. 10.1016/j.biomaterials.2011.10.059 22088888 PMC3278642

[B42] Wang H. J. Lin J. J. Lo W. Y. Chang C. P. Hsu C. H. Hsieh L. C. (2017). Clinical outcomes of polytetrafluoroethylene-covered stents for coronary artery perforation in elderly patients undergoing percutaneous coronary interventions. Acta Cardiol. Sin. 33, 605–613. 10.6515/ACS20170625A 29167613 PMC5694924

[B43] Weekes A. Bartnikowski N. Pinto N. Jenkins J. Meinert C. Klein T. J. (2022). Biofabrication of small diameter tissue-engineered vascular grafts. Acta Biomater. 15, 92–111. 10.1016/j.actbio.2021.11.012 34781026

[B44] Wu C. An Q. Li D. Wang J. Liping H. Chen H. (2014). A novel heparin loaded poly(l-lactide-co-caprolactone) covered stent for aneurysm therapy. Mater. Lett. 116, 39–42. 10.1016/j.matlet.2013.10.018

[B45] Yakub G. Toncheva A. Manolova N. Rashkov I. Danchev D. Kussovski V. (2016). Electrospun polylactide-based materials for curcumin release: photostability, antimicrobial activity, and anticoagulant effect. J. Appl. Polym. Sci. 133, 42940. 10.1002/app.42940

[B46] Zavan B. Gardin C. Guarino V. Rocca T. Cruz Maya I. Zanotti F. (2021). Electrospun PCL-based vascular grafts: *in vitro* tests. Nanomaterials 11, 751. 10.3390/nano11030751 33809791 PMC8002398

[B47] Zhou X. Pan Y. Liu R. Luo X. Zeng X. Zhi D. (2019). Biocompatibility and biodegradation properties of polycaprolactone/polydioxanone composite scaffolds prepared by blend or co-electrospinning. J. Bioact. Compatible Polym. 34, 115–130. 10.1177/0883911519835569

[B48] Zhu Y. Hu C. Li B. Yang H. Cheng Y. Cui W. (2013). A highly flexible paclitaxel-loaded poly(ε-caprolactone) electrospun fibrous-membrane-covered stent for benign cardia stricture. Acta Biomater. 9, 8328–8336. 10.1016/j.actbio.2013.06.004 23770223

